# Comprehensive evaluation of genetic and acquired thrombophilia markers for an individualized prediction of clinical thrombosis in patients with lymphoma and multiple myeloma

**DOI:** 10.1007/s11239-024-02977-0

**Published:** 2024-04-27

**Authors:** Irene Sánchez Prieto, Isabel Gutiérrez Jomarrón, Celia Martínez Vázquez, Pedro Rodríguez Barquero, Paula Gili Herreros, Julio García-Suárez

**Affiliations:** Hematology Department, Hospital Universitario Príncipe de Asturias, Alcalá de Henares, Madrid, Spain

**Keywords:** Cancer-associated thrombosis, Genetic thrombophilia variants, Lymphoma, Multiple myeloma, Prothrombotic biomarkers, Venous thromboembolism

## Abstract

Patients diagnosed with lymphoma or multiple myeloma are at elevated risk of venous thromboembolism (VTE). Optimum risk stratification and effective thromboprophylaxis can only be achieved through the development of a multiple-specific risk score that successfully captures all aspects of the heterogeneous prothrombotic environment existing in these patients. Our aim was to identify risk factors for thrombosis and suggest an improved tool combining clinical data, thrombo-inflammatory biomarkers and genetic (Thrombo inCode® test) variables for predicting thrombotic risk in patients with lymphoma and multiple myeloma. A prospective longitudinal study was conducted on newly-diagnosed lymphoma and multiple myeloma patients who presented at our institution between February 2020 and January 2021. The study included 47 patients with lymphoma and 16 patients with multiple myeloma. We performed a follow-up of 1 year or until September 2021. The incidence of venous thrombosis and associated risk factors were analysed, including the genetic Thrombo inCode® test. Khorana and ThroLy scores for lymphoma patients and IMPEDE VTE score for myeloma patients were calculated. At a median follow-up of 9.1 months, VTE incidence was 9.5% (6/63), with 4 and 2 patients with lymphoma and myeloma who developed the events, respectively. Univariate analysis showed that the incidence of thrombosis was significantly higher in patients with ECOG ≥ 2 and prior immobility. Median factor VIII levels were significantly higher in patients with thrombosis (with increased values in all of them). Moreover, there was a trend in genetic variant rs5985 (factor XIII) as a protective factor, and a trend to higher thrombotic risk in patients with factor V Leiden, rs2232698 variant (serpinA10), low total protein S activity, elevated D-dimer, aggressive lymphoma and treatment with dexamethasone. The results of our study demonstrate promise for the potential use of widely accessible markers to increase precision in risk prediction for VTE in patients with lymphoma and multiple myeloma, particularly ECOG ≥ 2, immobility and higher factor VIII levels, as well as lymphoma aggressiveness, treatment with dexamethasone and the haemostatic biomarkers D-dimer and total protein S activity. Additionally, genetic variants factor V Leiden, serpinA10 rs2232698 and factor XIII-A Val34Leu warrant further investigation for use in the research setting.

**Graphical Abstract:**

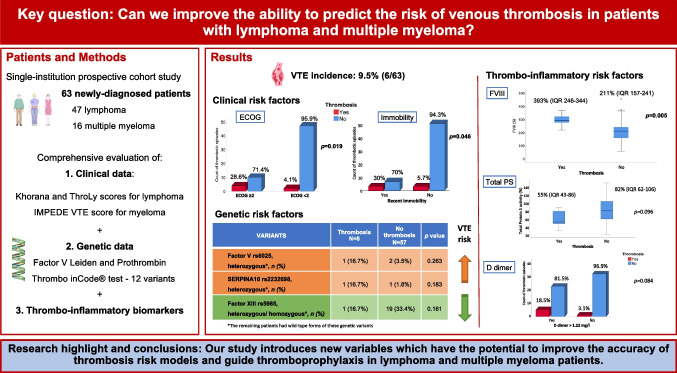

**Supplementary Information:**

The online version contains supplementary material available at 10.1007/s11239-024-02977-0.

## Introduction

Patients with cancer have a 4–eightfold greater risk of experiencing VTE compared to the general population [1], being the second cause of death in cancer patients. It also causes worsened patients’ morbidity, a delay in planification of systemic therapies and an increase of health-care costs [2]. Patients are exposed to a higher risk of thrombotic complications during the first months after cancer diagnosis and start of antineoplastic therapy [1, 3]. The incidence of VTE varies according to patient-related, disease-related and treatment-related factors [2], with an estimated heritability of about 60% [4]. In lymphoma, the rate of thrombosis ranges from 1.5% up to 59.5%. The basis of this variability probably lies in the heterogeneity of aggressiveness, tumour burden and location of the different types of lymphomas, with a demonstrated higher risk in patients with aggressive non-Hodgkin lymphoma (NHL) than in those with indolent NHL or Hodgkin lymphoma (HL) [1]. The incidence of thrombosis in patients diagnosed with multiple myeloma (MM) is 8.7 per 1000 person-years [5], with a greater risk in patients treated with immunomodulatory agents (IMiDs), especially in combination with dexamethasone or anthracyclines [6].

Thromboembolic complications can be prevented by primary thromboprophylaxis, which is included as a recommendation in guidelines for VTE management in cancer patients. However, pharmacological thromboprophylaxis increases the risk of haemorrhage, especially in certain populations (e.g. patients with thrombocytopenia). In ambulatory patients, it is recommended to be cautious and to identify patients who are at higher risk by using risk assessment models (RAMs), so that thromboprophylaxis is justified in certain cases [7]. The most consolidated predictive model in cancer patients is Khorana score, which is mainly focused on solid tumours [8]. More recently, other clinical RAMs have been proposed in hematological patients, such as ThroLy [9] in lymphoma and IMPEDE VTE [10] in MM. However, it remains unclear whether these RAMs should be generally recommended for identification of patients who are at major risk of thrombosis [7]. In order to approach this unmet need, novel scores including clinical and genetic variables have been developed and have shown promising results. These scores are TiC-Onco [11] and ONCOTHROMB [12] for patients with solid tumours, and TiC-LYMPHO [13] for lymphoma patients.

In the present work, we hypothesized that a personalized approach to the thrombotic risk based on the inclusion of genetic and acquired thrombophilia parameters in predictive scores could better identify those patients diagnosed with lymphoma or MM who would benefit most from thromboprophylaxis. The aim of this study was to analyse the incidence of venous thrombosis in a real-world cohort of lymphoma and MM patients and to identify clinical, laboratory or genetic variables that could potentially increase their thrombotic risk. Our results suggest that a new risk score that includes a combination of clinical, prothrombotic biomarkers and genetic (Thrombo inCode®- TiC) variables could be developed in order to better predict which patients should receive primary thromboprophylaxis.

## Methods

### Study design and participants

This is a prospective, longitudinal study that includes patients over 18 years of age diagnosed with lymphoma (based on the World Health Organization 2016 classification) [14] or MM (based on the 2014 International Myeloma Working Group updated criteria [15]) between February 2020 and June 2021. Follow-up was performed during the next 12 months following cancer diagnosis or until September 2021. For the analysis, lymphomas were classified according to the histological diagnosis and NHL were grouped according to their clinical aggressiveness into indolent and aggressive. Aggressive lymphomas included diffuse large B-cell lymphoma, high grade B-cell lymphoma, mantle cell lymphoma, T-cell lymphomas and follicular lymphomas grade 3B. Indolent lymphomas included marginal zone lymphoma, lymphoplasmacytic lymphoma and follicular lymphoma grade 1-3A. Patients diagnosed with monoclonal gammopathy of undetermined significance, chronic lymphocytic leukemia/small lymphocytic lymphoma or cutaneous lymphomas with no systemic manifestation were excluded.

This study was approved by the local ethics committee and performed in accordance with the ethical standards as laid down in the 1964 Declaration of Helsinki and its latter amendments. Informed consent was obtained from all patients before inclusion in the study.

### Follow-up and diagnosis of thromboembolic events

VTE symptoms were evaluated at baseline and regular follow-up visits throughout the first year. Apart from routine visits, the electronic health record was reviewed at 3, 6 and 12 months from disease diagnosis. Research team analysed all emergent visits and hospital admissions. When a thrombotic episode occurred, this was appropriately registered.

There was no routine screening for VTE. Objective imaging methods were performed to confirm or exclude the diagnosis only when a patient developed symptoms of VTE. Duplex ultrasound was applied for diagnosis of deep vein thrombosis (DVT), and spiral computed tomography scan was applied for diagnosis of pulmonary embolism (PE).

### Sample genotyping

DNA was obtained from blood samples extracted at the time of diagnosis. These were genotyped using the *Thrombo inCode kit* (Gen inCode), a real-time PCR method that analyses 12 single nucleotide polymorphisms (SNPs) known to be associated to VTE (Table S1 in Supplementary Material) [4]. A score related to thrombosis probability was calculated through TiC test, based on clinical and genetic variables.

### Prothrombotic biomarkers

At the time of cancer diagnosis, blood samples were obtained in order to analyse the laboratory parameters, including blood cell counts and D-dimer, fibrinogen and inflammatory markers values (C reactive protein – CRP, lactate dehydrogenase – LDH, albumin). Acquired/ Plasmatic thrombophilia testing consisted of: lupus anticoagulant, anticardiolipin IgG and IgM antibodies, levels of coagulation factors VIII (FVIII) and XII, activity levels of natural anticoagulant proteins (protein S, protein C and antithrombin III) and free protein S levels. Patients who developed a thrombotic event at the same time of cancer diagnosis were excluded for analysis of plasmatic thrombophilia. Later on, samples were extracted at 3, 6 and 12 months from diagnosis, with fibrinogen and D dimer testing. Moreover, in patients who developed venous thrombosis, blood cell counts, D dimer and fibrinogen were measured at this time.

### Clinical risk factors

Data for demographic and clinical variables that could potentially be associated with VTE were extracted at diagnosis, based in previous studies concerning lymphoma and MM patients [9, 10, 13, 16]. These included patient characteristics (age, sex, race, personal or family history of thrombosis, body mass index (BMI), ECOG performance status, comorbidities, recent (< 6 weeks) immobility period, hospitalization in the last 12 weeks, acute infection, rheumatologic disorder, baseline antithrombotic therapy and type of drug used), lymphoma characteristics (histological type, stage, bulky disease, mediastinal or central nervous system – CNS – involvement, extranodal disease and B symptoms) and multiple myeloma characteristics (paraprotein secretion and type of protein involved, International Scoring System (ISS) stage and pelvic, hip or femur fracture). In this study, race was classified as White, Black, American Indian/ Alaska Native or Asian Pacific Islander according to SEER Race Recode [17]. Treatment variables were also recorded at diagnosis and updated during follow-up: use of recombinant erythropoietin (EPO), steroids (type of drug and dosage), anthracyclines, cyclophosphamide, lenalidomide, bortezomib or central venous catheter (CVC) (mainly peripherally-inserted central venous catheter (PICC) in our series).

### Performance of predictive scores

At the time of diagnosis, Khorana algorithm was performed for patients with lymphoma, and ThroLy score was tested in the same subpopulation after 1 cycle of antineoplastic therapy. In patients with MM, IMPEDE VTE was performed at cancer diagnosis. Patients who developed a thrombotic event at the same time of cancer diagnosis were excluded for this analysis.

### Statistical analysis

Continuous variables were recorded as median (interquartile range) and categorical variables as proportions. Univariate association between either clinical, laboratory or genetic variables and thrombosis was determined by T-test or Mann–Whitney U test for continuous variables and X^2^ or Fisher tests for categorical variables. The correlation between two continuous variables was analysed with Pearson’s r or Spearman rank correlation coefficients. For categorical variable D-dimer, ROC (Receiver Operating Characteristic) curves were used to select the cut-point that maximizes sensitivity and specificity (D-dimer 1.22 mg/L). Multiple comparisons between paired continuous variables were performed with paired T-test or Wilcoxon signed-rank test, and Holm method was used for *p* value correction. All reported *p* values were 2-sided and were considered significant at the 5% significance level. The statistical analysis was performed using IBM SPSS Statistics software (version 25.00) (IBM Corp., Armonk, NY, USA) [18]. The score derived from the TiC test was calculated by multivariate logistic regression analysis of clinical and genetic variables, using the *Thrombo inCode kit* application [19].

## Results

### Patients characteristics

The study population included 47 (74.6%) patients diagnosed with lymphoma and 16 (25.4%) patients diagnosed with MM. The median patient age was 64 years (interquartile range (IQR), 51–72 years), with 36 (57.1%) men. Patients’ race distribution was: 62 White and 1 American Indian. Twenty-two (34.9%) patients were on antithrombotic therapy at baseline: 15 (23.8%) were on anticoagulant drugs, one of them on a therapeutic dose, 4 (6.3%) were on antiplatelet drugs and 3 (4.8%) were on both. The most common reason for antithrombotic therapy was the initiation of lenalidomide in 8 (36.4%) patients, all of whom were diagnosed with MM. Among patients diagnosed with lymphoma, the majority (87.2%) had NHL, with 41 cases, and the majority of these were high-grade NHL (70.7%). Ann-Arbor stage was ≥ 3 in 30 (63.8%) patients and no CNS involvement was observed. Among patients diagnosed with MM, the majority had ISS III (62.5%) and 7 (43.8%) had IgG isotype. Other characteristics of the population with and without VTE are shown in Table 1 and Tables S2 and S3 in Supplementary Material.

**Table 1:** Characteristics of the patients with and without thrombosis

	Thrombosis	No thrombosis	*p* value
	N = 6	N = 57	
Age (years), *median (IQR)*	69 (60–70)	64 (50–73)	0.623
Sex (female), *n (%)*	4 (66.7%)	23 (40.3%)	0.388
Initial thromboprophylaxis, *n (%)*	2 (33.3%)	20 (35.1%)	> 0.999
Clinical risk factors for thrombosis, *n*	N = 6	N = 57	
Family history of thrombosis, *n (%)*	2 (33.3%)	16 (28.1%)	> 0.999
Personal history of thrombosis, *n (%)*	0 (0%)	8 (14%)	> 0.999
BMI ≥ 30 kg/m^2^, *n (%)*	2 (33.3%)	10 (17.5%)	0.320
Diabetes, *n (%)*	2 (33.3%)	10 (17.5%)	0.320
Hypertension, *n (%)*	1 (16.7%)	28 (49.1%)	0.205
Hyperlipidemia, *n (%)*	4 (66.7%)	15 (26.3%)	0.062
Recent hospitalization, *n (%)*	1 (16.7%)	4 (7%)	0.404
Acute infection and/ or rheumatologic disorder, *n (%)*	1 (16.7%)	3 (5.3%)	0.337
Recombinant erythropoietin^a^, *n (%)*	1 (25%)	17 (31.5%)	> 0.999
Central venous catheter^a^, *n (%)*	2 (50%)	16 (29.6%)	0.581
Steroids^a^, *n*	N = 4	N = 45	
Dexamethasone, *n (%)*	3 (75%)	23 (51.1%)	0.612
Other steroids, *n (%)*	1 (25%)	22 (48.9%)	
Lenalidomide^a, b^, *n (%)*	0 (0%)	12 (22.2%)	0.571
Bortezomib^a^, *n (%)*	1 (25%)	13 (24.1%)	> 0.999
Anthracyclines^a^, *n (%)*	3 (75%)	32 (59.3%)	> 0.999
Cyclophosphamide^a^, *n (%)*	4 (100%)	37 (68.5%)	0.310
Plasmatic thrombophilia, *n*	N = 5	N = 56	
Factor VIII > 200%, *n (%)*	5 (100%)	32 (57.1%)	0.147
Reduced total protein S activity^c^, *n (%)*	3 (60%)	19 (33.9%)	0.341
Reduced free protein S^c^, *n (%)*	1 (20%)	11 (19.6%)	> 0.999
Protein C activity < 70%, *n (%)*	0 (0%)	2 (3.8%)	> 0.999
Antithrombin III activity < 80%, *n (%)*	0 (0%)	2 (3.8%)	> 0.999
Factor XII < 60%, *n (%)*	0 (0%)	1 (1.8%)	> 0.999
Positive lupus anticoagulant,* n (%)*	0 (0%)	6 (10.7%)	> 0.999
Anticardiolipin antibodies, *n (%)*			
IgG IgM Negative	0 (0%) 0 (0%) 5 (100%)	4 (7.1%) 1 (1.8%) 51 (91.1%)	> 0.999
D-dimer, *n*	N = 6	N = 53	
D-dimer > 1.22 mg/L, *n (%)*	5 (83.3%)	22 (41.5%)	0.084
Fibrinogen > 400 mg/dL *(%)*	4 (66.7%)	24 (42.1%)	0.396
Platelets > 350 × 10^3^/μL, *n (%)*	2 (33.3%)	9 (15.8%)	0.280
C reactive protein > 10 mg/L, *n (%)*	5 (83.3%)	30 (52.6%)	0.220
Albumin < 3,2 g/dL, *n (%)*	2 (33.3%)	10 (17.5%)	0.320
SNPs, risk alleles^d^, *n*	N = 6	N = 57	
Factor V rs6025, *n (%)*			
0 Risk Alleles 1 Risk Allele	5 (83.3%) 1 (16.7%)	55 (96.5%) 2 (3.5%)	0.263
SERPINA10 rs2232698, *n (%)*			
0 Risk Alleles 1 Risk Allele	5 (83.3%) 1 (16.7%)	56 (98.2%) 1 (1.8%)	0.183
Factor XIII rs5985, *n (%)*			
0 Risk Alleles 1 Risk Allele 2 Risk Alleles	5 (83.3%) 0 (0%) 1 (16.7%)	38 (66.7%) 16 (28.1%) 3 (5.3%)	0.161
Factor II rs1799963, *n (%)*			
0 Risk Alleles 1 Risk Allele	6 (100%) 0 (0%)	55 (96.5%) 2 (3.5%)	> 0.999
Factor XII rs1801020, *n (%)*			
0 Risk Alleles 1 Risk Allele 2 Risk Alleles	5 (83.3%) 1 (16.7%) 0 (0%)	35 (61.4%) 21 (36.8%) 1 (1.8%)	0.465
A1 blood group, *n* (%)	2 (33.3%)	17 (29.8%)	> 0.999

*IQR* interquartile range, *BMI* body mass index, *SNPs* single nucleotide polymorphisms

^a^For treatment variables, patients with thrombosis before the start of antineoplastic therapy and those who received no therapy during follow-up were excluded for this analysis (n = 4 for “Thrombosis” and n = 54 for “No thrombosis”)

^b^Patients were on prophylaxis with low molecular weight heparin

^c^Reduced total protein S activity < 55% for females and < 77% for males; reduced free protein S < 50% for females and < 70% for males

^d^No risk alleles for Factor V rs118203905, rs118203906 or Serpin C1 rs121909548 were found

### Thromboembolic events

**Fig. 1:**
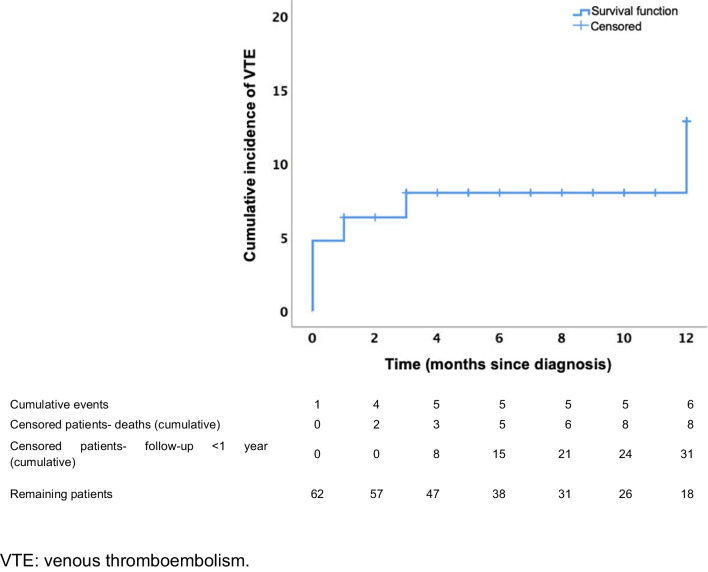
**Cumulative incidence of venous thrombosis**. During follow-up, the cumulative incidence of VTE was 9.5% (patients who died or could not complete 12 months of follow-up were censored at the time of death or when follow-up was interrupted)

At a median follow-up of 9.1 months (IQR 5.1–12), 6 (9.5%) patients developed VTE (Fig. 1), of which 4 had been diagnosed with lymphoma and 2 with myeloma. To date, after 3 years since the first patient was included, no other thrombotic episodes have appeared. In the majority of patients (5/6), thrombosis occurred during the first 3 months since cancer diagnosis, with a median time to thrombosis of 36 days (IQR 4–92). Two of the 6 patients experienced the event before the start of antineoplastic therapy. Detailed description of VTE episodes is shown in Table 2. Regarding laboratory markers, a majority of the patients had elevated fibrinogen (3/5) and D-dimer (3/4) values at the time of the episode, with a median of 443 mg/dL (IQR 366–512) and 0.51 mg/L (0.49–3.04), respectively.

**Table 2:** Detailed description of thrombotic episodes in each patient

	1	2	3	4	5	6
**Age (years)**	54	72	63	72	68	71
**Baseline antithrombotic therapy**	Prophylactic enoxaparin because of SARS-CoV-2 infection	ASA due to DM with organ damage. No history of CVD	No	No	No	No (prophylactic enoxaparin during recent hospitalization
**Type of thrombosis**	PE	DVT in lower leg	DVT in left internal jugular vein	DVT in popliteal vein	Catheterrelated DVT in right arm (PICC)	PE
**Time to thrombosis (days)**	4	365	0	92	29	44
**Additional risk factors for thrombosis**	SARS-CoV-2 infection Immobility	No other specific risk factors found	Aggressive malignancy	Immobility	PICC Recent hospitalization	Recent hospitalization with immobility
**Type of malignancy and status**	Active IgG MM, with no therapy started	DLBCL on remission, without treatment	Active HGBL, with no therapy started	T-cell lymphoma, under treatment	Active HGBL, under treatment	Active IgG MM, under treatment
**Time of thrombosis treatment (months)**	13	6	6.4	6.5	3.6	6.4
**Drug used for thrombosis treatment and dose**	Bemiparin 7500 IU daily	Bemiparin 7500 IU daily	Bemiparin 10,000 IU daily	Bemiparin 7500 IU daily	Bemiparin 5000 IU daily	Bemiparin 7500 IU daily, changed to apixaban 5 mg twice daily
**Resolution of thrombosis**	Yes	Yes	Yes	Yes	Yes	Yes

*ASA* acetylsalicylic acid, *DM* diabetes mellitus, *CVD* cardiovascular disease, *PE* pulmonary embolism, *DVT* Deep venous thrombosis, *PICC* peripherally-inserted central catheter, *MM* multiple myeloma, *DLBCL* diffuse large B-cell lymphoma, *HGBL* high-grade B lymphoma

The median duration of full-dose anticoagulation was 6.4 months (IQR 6.1–6.5). At this time, the majority (5/6) discontinued anticoagulation due to non-active cancer.

### Genetic analysis (Thrombo inCode®)

The TiC tool showed that median calculated genetic score was higher in the patients who developed VTE (1.86; IQR 0.70–8.30) than in those who did not (1.38; IQR 0.54–3.09); *p* = 0.470. The most frequent genetic variants were mutations in FXII (36.5%) and FXIII (31.7%) genes and presence of A1 haplotype (30.2%). FVL and SERPINA10 mutations were more prevalent in VTE patients compared to non-VTE patients. The heterozygous or homozygous mutated forms in FXIII gene were more frequent in patients who did not develop thrombosis than in those with events; none of the 16 patients who had the heterozygous mutation developed thrombosis (Table 1).

### Other risk factors for thromboembolism

**Fig. 2:**
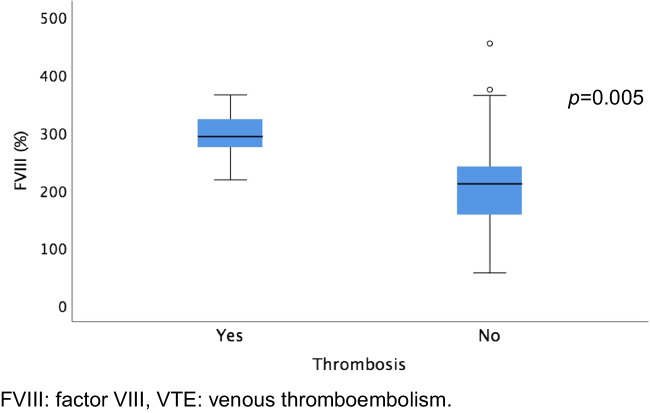
**Baseline factor VIII (%) in patients with and without thrombosis**. FVIII levels were significantly higher in patients who developed VTE compared to those who did not

Regarding haemostatic biomarkers, FVIII > 200% and low total PS activity were the most common abnormalities in the plasmatic thrombophilia study (60.7% and 36.1% of the population, respectively). In patients with thrombosis, elevated FVIII was present in 5 out of 5 cases, and reduced PS activity was present in 3 out of 5 cases (Table 1). Median FVIII levels were significantly higher in patients who developed VTE (393%; IQR 246–344) compared to those who did not (211%; IQR 157–241); *p* = 0.01. (Fig. 2). In addition, median total PS activity was lower in patients who developed VTE (55%; IQR 43–86) compared to those without VTE (82%; IQR 62–106); *p* = 0.096. There was a strong significant correlation between median total PS activity and median free PS (r = 0.787; *p* < 0.001). However, median free PS was not reduced in patients with thrombosis (91%; IQR 68–96) compared to those without events (86%; IQR 69–110); *p* = 0.577.

Furthermore, D-dimer and fibrinogen had high values more frequently in VTE group than in non-VTE patients (Table 1). We found that the median D-dimer concentration was slightly higher in patients who experienced VTE (1.96 mg/L; IQR 1.16–3.72) compared to those who did not (0.65 mg/L; IQR 0.39–2.03); *p* = 0.139. Fibrinogen levels were also slightly higher in the VTE group (median 473 mg/dL; IQR 317–556) compared to the non-VTE group (median 371 mg/dL; IQR 324–471); *p* = 0.332. Finally, patients with thrombosis exhibited abnormal results in values of platelets, CRP and albumin more frequently than non-VTE patients (Table 1).

Comparison of coagulation parameters at diagnosis and during follow-up showed significant differences between baseline D-dimer and D-dimer at 3, 6 and 12 months of follow-up (Table S4 in Supplementary Material). Other comparisons of coagulation parameters during follow-up did not show interesting results (data not shown).

**Fig. 3:**
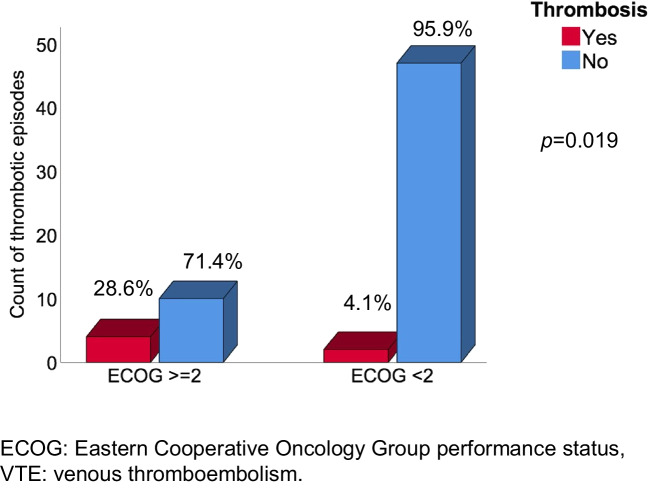
**Thrombosis in patients with ECOG ≥ 2 or ECOG < 2**. The incidence of VTE was compared according to the performance status at the time of malignancy diagnosis

Univariate regression analysis demonstrated that patients with an impaired performance status, measured by an ECOG ≥ 2, experienced VTE at a significantly higher frequency than patients with ECOG < 2 (Fig. 3). In the same way, patients with immobility in the 6 weeks prior to cancer diagnosis had thrombosis more frequently than the other patients (30% vs. 5.7%; *p* = 0.046).

Other clinical variables showed a tendency towards an increase in thrombotic risk, such as an older age, female sex or BMI ≥ 30 kg/m^2^ (Table 1). All of the patients diagnosed with lymphoma who developed thrombosis had aggressive NHL (100% vs. 67.6% for those without VTE) and Bulky disease was more frequent in thrombosis group than in non-VTE one (50% vs. 18.6%) (Table S2 in Supplementary Material). Regarding treatment characteristics in the whole population, more patients in the VTE group had a permanent CVC (50% vs. 29.6%) or received steroids (100% vs. 83.3%), especially dexamethasone, anthracyclines (75% vs. 59.3%) or cyclophosphamide (100% vs. 68.5%). On the contrary, antithrombotic prophylaxis was not associated with a lower VTE incidence compared to patients with no thromboprophylaxis (9.8% vs. 9.1%) (Table 1). In the same way, patients diagnosed with MM who received lenalidomide-based therapies did not show a higher risk of VTE compared to those who received other treatments (data not shown).

Finally, the RAMs analysed (Khorana, ThroLy and IMPEDE VTE) did not manage to adequately predict thrombotic risk in our population (Table S5 in Supplementary Material).

## Discussion

An integrated evaluation of multiple risk factors is likely to be the standard for multifactorial diseases such as cancer-associated thrombosis (CAT). To the best of our knowledge, our study is the first to conduct a prospective and comprehensive study of several clinical, thrombo‐inflammatory, and genetic variables associated with VTE to identify lymphoma and MM patients at risk for CAT. Our findings show the risk contribution of two clinical risk factors, namely ECOG ≥ 2 and immobility in the 6 weeks prior to the diagnosis of malignancy, as well as increased levels of FVIII. The presence of a low total PS activity and two genetic variants, namely factor V Leiden and rs2232698 (serpinA10), showed a tendency to a higher incidence of thrombosis. Interestingly, the genetic variant rs5985 (factor XIII) was found to have a slight protective effect against thrombosis. Our study introduces new variables which have the potential to improve the accuracy of thrombosis risk models and guide thromboprophylaxis in lymphoma and MM patients.

The incidence of VTE in the present population at 9 months of follow-up was 8.5% and 12.5% in patients diagnosed with lymphoma and MM, respectively; these figures are consistent with the range reported in a recent review for the same follow-up period (20). This finding supports the observation that the highest incidence of VTE occurs within the first 3 months after cancer diagnosis [9, 20, 21], which can be attributed to the higher tumour burden and initiation of antineoplastic treatment [3, 9]. Notably, all VTE events among patients with lymphoma occurred in patients diagnosed with aggressive lymphomas, as previously reported [22]. This suggests that the lymphoma aggressiveness is a major factor contributing to the risk of VTE.

Corresponding to several previous findings [9, 23–26], clinical RAMs (Khorana and ThroLy scores in lymphoma, and IMPEDE VTE in MM) have not demonstrated satisfactory thrombotic prediction performance in our patients with lymphoma and MM. One possible explanation to this could be the high rate of antithrombotic prophylaxis in our patients. Despite the lack of association between thromboprophylaxis and VTE in our population, we cannot firmly conclude that thromboprophylaxis had no influence in preventing thrombosis and in the prediction performed by the RAMs. Therefore, optimum risk stratification can only be achieved through the development of a lymphoma and MM-specific risk score that can successfully capture all aspects of the heterogeneous prothrombotic environment that exists in these patients.

Among the strengths of this study is the comprehensive analysis of 12 genetic variants that have been previously identified in genome-wide analysis as being associated with VTE in both the general population and patients with solid tumours [4, 11, 12]. Consistent with the findings of previous studies conducted in patients with solid tumours [11], our study also found that patients with lymphoma and MM who had two specific genetic variants (factor V Leiden and SERPINA10 variants) had a higher incidence of VTE. In line with our results, factor V Leiden has previously demonstrated to confer a higher thrombotic risk in oncological patients, having a synergistic interaction with cancer [27]. The prothrombotic mechanism of this variant is explained by the conformational change in the binding site between factor V and activated PC (aPC), increasing resistance of activated factors V and VIII to degradation by aPC ([28]. Moreover, cancer could additionally increase thrombotic risk, by generating an acquired resistance to aPC [29].

Regarding SERPINA10 mutation, a threefold risk of developing VTE has been demonstrated in a prior investigation [30]. The proposed mechanism for this is the impaired function of SERPINA10 molecule as potent inhibitor of activated factors X and XI [30, 31].

Our study supports the findings of a meta-analysis [32], and suggests that the FXIII-A Val34Leu mutation, which is present in approximately 25% of European Caucasians, provides a slight protective effect against VTE. The proposed mechanism for this effect is interesting. In plasmas with normal fibrinogen levels, the Leu34 allele produces clots with thinner fibers and decreased permeability, whereas in plasmas with high fibrinogen, it produces clots with thicker fibers, and increased permeability and susceptibility to fibrinolysis [33]. These observations indicate that both FXIII genotype and plasma fibrinogen concentration should be considered when calculating thrombosis risk in population studies.

Emerging evidence suggests that there are cancer-type-specific haemostatic biomarkers of VTE [34–36]. Our analysis revealed that lymphoma and MM patients with VTE had higher levels of D-dimer and FVIII, as well as a lower total PS activity, overall suggesting a predominantly thrombotic state. Moreover, we have observed that almost 80% of our patients displayed one of these hypercoagulation abnormalities. This subset of patients did indeed exhibit a higher rate of thrombosis.

These findings are in accordance with a recent review on the pathophysiological mechanisms underpinning thrombosis in untreated lymphoma and leukemia patients with active disease, which concluded that these patients appear to display a hypercoagulable phenotype including significant elevations in FVIII and D-dimer levels and reduced protein S levels. The etiology of these haemostatic abnormalities is unclear, with possible causative mechanisms including a combination of chronic endothelial activation and dysfunction, increased bone marrow angiogenesis, and disturbances in the VWF/ADAMTS‐13 axis [37]. Other potential contributors to the hypercoagulable profile seen in lymphoma and MM patients include elevations in fibrinogen levels. However, the relative effect and overall contribution of this abnormality towards VTE occurrence in lymphoma and MM patients has not yet been fully determined [38, 39]. The use of these haemostatic biomarkers for prediction of VTE in patients with lymphoma and MM warrants further investigation in prospective trials [40, 41].

Importantly, in our study, an impaired performance status measured by an ECOG ≥ 2 and a period of immobility in the 6 weeks prior to malignancy diagnosis were significantly associated to VTE. Similarly, several studies reported an ECOG > 1 or bed rest for more than 3 days as risk factors for thrombosis in patients with lymphoma or MM [9, 13, 42, 43]. A potential explanation for this is that a reduction of the daily activity, with partial or complete immobilization, could promote the deceleration of blood flow in the venous bed [42]. Other clinical factors that in our investigation showed a tendency to higher thrombotic risk (bulky disease in lymphoma patients and treatment with dexamethasone) have already been associated to an increased thrombotic risk in prior publications from lymphoma and MM populations [10, 42, 44].

We acknowledge limitations of our study. We recognize that this is a pilot study with a small number of patients, and due to this, a proportional low number of thrombotic episodes. As a consequence, we could not manage to perform a multivariate analysis to confirm the associations found or other associations between the risk factors included and thrombosis. The difficulty in the comparison of our results with respect to other works is possibly derived from the small size of our cohort. Moreover, haemostatic biomarkers have important limitations for clinical implementation as of their low specificity.

## Conclusion

From the aforementioned data, adjunctive clinical risk factors, biomolecular markers, and genetic variants assessment could all ameliorate VTE prediction, while the introduction of novel computational analyses could help with gaining knowledge from available datasets to obtain accurate and precise personalized risk estimates in lymphoma and MM patients. Our data indicate that prediction of VTE in lymphoma and MM patients may be more accurate if a limited set of genetic predictors (FV rs6025, serpinA10 rs2232698 and FXIII-A Val34Leu), clinical predictors (ECOG, immobilization, the aggressiveness of the neoplasia and treatment with dexamethasone), and haemostatic biomarkers (D-dimer, FVIII and total PS activity) are included in a new risk score, which could contribute to personalized, risk-stratified patient management in the future. The results could change clinical practice and have an important impact in national health systems.

### Supplementary Information

Below is the link to the electronic supplementary material.

Supplementary file1 (DOCX 17 KB)

## Data Availability

The datasets presented in this article are not openly available because the data are part of an ongoing study at this centre.
