# Supplementary material for: Comprehensive evaluation of genetic and acquired thrombophilia markers for an individualized prediction of clinical thrombosis in patients with lymphoma and multiple myeloma
Source: J Thromb Thrombolysis. 2024 Apr 27;57(6):984–95. doi: 10.1007/s11239-024-02977-0 (PMC11315779; doi:10.1007/s11239-024-02977-0)
Supplement: Supplementary file 1 — Supplementary file1 (DOCX 17 KB) [file 11239_2024_2977_MOESM1_ESM.docx]

**SUPPLEMENTARY MATERIAL**

**Table S1. Genetic variants included in our analysis, based on the Thrombo inCode^®^ test** (Adapted from: Soria, 2014) [4].

| **SNP** | **Gene** | **Mutation** |
| --- | --- | --- |
| rs6025- Factor V Leiden | Factor V | R506Q |
| rs118203905- Factor V Hong Kong | Factor V | R306G |
| rs118203906- Factor V Cambridge | Factor V | R306T |
| rs1799963 | Factor II/ Prothrombin | G20210A |
| rs1801020 | Factor XII | C46T |
| rs5985 | Factor XIII | V34L |
| rs121909548 | SerpinC1 | A384S |
| rs2232698 | SerpinA10 | R67X |
| rs7853989 | ABO | A1 carriers |
| rs8176719 |  |  |
| rs8176743 |  |  |
| rs8176750 |  |  |

SNP, Single nucleotide polymorphism.

**Table S2. Clinical lymphoma characteristics in patients with or without thrombosis.**

|  | **Thrombosis**  ***N=4*** | **No thrombosis**  ***N=43*** | ***p* value** |
| --- | --- | --- | --- |
| Histogical type, *n (%)*:  DLBCL/ HGBL/ FL IIIB  T-cell lymphoma  Mantle cell lymphoma  FL ≤IIIA  Marginal zone lymphoma  Lymphoplasmacytic lymphoma  Hodgkin lymphoma | 3 (75%)  1 (25%)  0 (0%)  0 (0%)  0 (0%)  0 (0%)  0 (0%) | 21 (48.8%)  1 (2.3%)  4 (9.3%)  8 (18.6%)  2 (4.7%)  1 (2.3%)  6 (14%) | 0.301 |
| Aggressive NHL^a^, *n (%)* | 4 (100%) | 25 (67.6%) | 0.302 |
| Bulky disease, *n (%)* | 2 (50%) | 8 (18.6%) | 0.194 |
| B symptoms, *n (%)* | 2 (50%) | 19 (44.2%) | >0.999 |
| Ann-Arbor, *n (%)*:  I  II  III  IV | 0 (0%)  3 (75%)  0 (0%)  1 (25%) | 3 (7%)  11 (25.6%)  3 (7%)  26 (60.4%) | 0.328 |
| Extranodal disease, *n (%)* | 1 (25%) | 22 (51.2%) | 0.609 |
| Mediastinal involvement, *n (%)* | 1 (25%) | 17 (39.5%) | >0.999 |

DLBCL, diffuse large B-cell lymphoma; HGBL, high-grade B-cell lymphoma; FL, follicular

lymphoma; NHL, non-Hodgkin lymphoma.

^a^Total NHL: n=4 for “Thrombosis” and n=37 for “No thrombosis”.

**Table S3. Clinical multiple myeloma characteristics in patients with or without thrombosis.**

|  | **Thrombosis**  ***N=2*** | **No thrombosis**  ***N=14*** | ***p* value** |
| --- | --- | --- | --- |
| Multiple myeloma, *n (%)*:  IgG  Bence-Jones  IgA  IgM | 2 (100%)  0 (0%)  0 (0%)  0 (0%) | 5 (35.7%)  5 (35.7%)  3 (21.4%)  1 (7.2%) | 0.708 |
| ISS, *n (%)*:  I  II  III | 0 (0%)  1 (50%)  1 (50%) | 2 (14.3%)  3 (21.4%)  9 (64.3%) | 0.625 |

ISS, International Staging System.

**Table S4. Comparison of D-dimer values at diagnosis with values at 3, 6 and 12 months.**

| **D-DIMER (mg/L)** | **Median (IQR)** | ***p* value** | **Corrected *p* value** |
| --- | --- | --- | --- |
| Baseline vs. 3 months | 0.81 (0.44-2.10) vs. 0.55 (0.41-1.34) | 0.001 | 0.005 |
| Baseline vs. 6 months | 0.81 (0.44-2.10) vs. 0.41 (0.32-0.62) | 0.001 | 0.006 |
| Baseline vs. 12 months | 0.81 (0.44-2.10) vs. 0.43 (0.29-0.73) | 0.002 | 0.008 |

IQR, interquartile range.

**Table S5. Thrombosis according to high, intermediate and low risk groups classified by predictive scores in patients with lymphoma and myeloma.**

|  | **Thrombosis**  **n (%)** | **No thrombosis**  **n (%)** | ***p value*** |
| --- | --- | --- | --- |
| **LYMPHOMA** | | | |
| **Khorana (n=46)**  High risk  Intermediate risk | 1 (16.7%)  2 (5%) | 5 (83.3%)  38 (95%) | 0.349 |
| **ThroLy (n=43)**  High risk  Intermediate risk  Low risk | 0 (0%)  2 (10.5%)  1 (9.1%) | 13 (100%)  17 (94.4%)  10 (90.9%) | 0.715 |
| **MULTIPLE MYELOMA** | | | |
| **IMPEDE VTE (n=16)**  High risk  Intermediate risk  Low risk | 1 (50%)  0 (0%)  1 (10%) | 1 (50%)  4 (100%)  9 (90%) | 0.292 |
